# Depressive symptoms among adults in Germany

**DOI:** 10.17886/RKI-GBE-2017-070

**Published:** 2017-10-09

**Authors:** Julia Bretschneider, Ronny Kuhnert, Ulfert Hapke

**Affiliations:** Robert Koch Institute, Department of Epidemiology and Health Monitoring, Berlin

**Keywords:** PREVALENCE, DEPRESSIVE SYMPTOMS, DEPRESSION, HEALTH MONITORING, GERMANY

## Abstract

Depressive symptoms imply a loss of quality of life, leading to increased morbidity and mortality as well as increased costs to the healthcare system. Information on the prevalence and distribution of depressive symptoms in the population is essential in light of planning prevention and care. GEDA 2014/2015-EHIS surveyed current depressive symptoms among adults in Germany through the Patient Health Questionnaire (PHQ-8). The surveyed prevalence of 10.1% indicates the widespread occurrence of depressive symptoms, regardless of an actual clinical diagnosis of depression. Prevalence for women (11.6%) is higher than for men (8.6%). Further differences exist concerning age and education as well as regional differences. The results are discussed in the light of the data available so far.

## Introduction

Depression is one of the most common mental health disorders and is related to a high disease burden both for individuals suffering from the condition as also for society as a whole [1-3]. German social insurance carriers have recorded a significant increase in the role of depression in health services over the past few years, a process which has been accompanied by increasing public awareness [4-6].

The term depression covers a heterogeneous spectrum of depressive disorders which can be categorised for example with regard to the severity and course of depressive symptoms [7]. For a physician to diagnose depression requires, beyond simply confirming the presence of depressive symptoms, the fulfilment of specific diagnostic criteria, such as the presence of certain key symptoms. However, depressive symptoms such as despondency and loss of energy can also affect persons that do not fulfil the necessary clinical diagnostic criteria of depression and for example experience subthreshold depression, or experience such symptoms yet in the context of other mental and physical disorders. Among these are depressive symptoms related to emotionally stressful life events, substance abuse (alcohol, drugs, etc.) and chronic physical illness or as side effects of certain medications. Regardless of their diagnostic classification, depressive symptoms lead to a subjective deterioration in health and health-related quality of life [8, 9] and are associated with increased morbidity and mortality rates [10, 11]. Moreover, depressive symptoms increase the number of outpatient physician visits and sick leave days [12] and in particular in older age lead to increasing health service utilisation [13].

Epidemiological estimates on the prevalence and distribution of depression symptoms in the population are therefore highly relevant to public health and are an important basis for developing prevention and healthcare services. Health monitoring at the Robert Koch Institute initially collected prevalence data for the adult population in Germany in the German Health Interview and Examination Survey for Adults (DEGS1, 2008-2011) [14] and we now report the corresponding data from GEDA 2014/2015-EHIS.

**GEDA 2014/2015-EHIS**

**Data holder:** Robert Koch Institute

**Aims:** To provide reliable information about the population’s health status, health-related behaviour and health care in Germany, with the possibility of a European comparison

**Method:** Questionnaires completed on paper or online

**Population:** People aged 18 years and above with permanent residency in Germany

**Sampling:** Registry office sample; randomly selected individuals from 301 communities in Germany were invited to participate

**Participants:** 24,016 people (13,144 women; 10,872 men)

**Response rate:** 26.9%

**Study period:** November 2014 - July 2015

**Data protection:** This study was undertaken in strict accordance with the data protection regulations set out in the German Federal Data Protection Act and was approved by the German Federal Commissioner for Data Protection and Freedom of Information. Participation in the study was voluntary. The participants were fully informed about the study’s aims and content, and about data protection. All participants provided written informed consent.

More information in German is available at

www.geda-studie.de

## Indicator

GEDA 2014/2015-EHIS surveyed current depressive symptoms through a self-administered paper-based or online questionnaire. The survey applied a German version of the 8-item depression module of the Patient Health Questionnaire (PHQ-8) to evaluate symptoms of a major depressive disorder (but did not consider suicidal thoughts) in accordance with the DSM-IV manual (Diagnostic and Statistical Manual of Mental Disorders, 4th edition) regarding the presence and frequency of symptoms during the past two weeks [9, 15]: Depressed mood or irritable, decreased interest or pleasure, significant weight change or change in appetite, change in sleep, psychomotor agitation or retardation, fatigue or loss of energy, guilt/worthlessness, diminished ability to concentrate. Each of these eight items was rated on a scale between 0 (not at all), 1 (several days), 2 (more than half of the days) and 3 (nearly every day). Sum values between 10 and 24 indicate current depressive symptoms [9]. Prevalence in the following section is presented with 95% confidence intervals (95% CI) stratified by age, gender and educational level as well as federal state. Differences between these groups are interpreted as statistically significant if the respective confidence intervals do not overlap.

The analyses are based on data from 23,602 participants aged 18 years and older (12,900 women and 10,702 men) with valid PHQ-8 data. The calculations were carried out using a weighting factor that corrects for deviations within the sample from the German population (as of 31 December 2014) with regard to gender, age, district type and educational level. The International Standard Classification of Education (ISCED) was used to classify the responses provided on educational level [16]. A detailed description of the methodology used in the GEDA 2014/2015-EHIS study can be found in Lange et al. 2017 [17] as well as in the article German Health Update: New data for Germany and Europe, which was published in Issue 1/2017 of the Journal of Health Monitoring.

## Results and discussion

The prevalence of current depressive symptoms among adults in Germany is 10.1% (Table 1). Prevalence among women (11.6%) is significantly higher than among men (8.6%). Gender differences are recorded across all age groups. Significant differences also exist with regard to age: Among women aged 18 to 29, prevalence is 16.4% and therefore higher than among older women. The lowest frequencies are recorded for women and men aged 65 and over (women 8.7%; men 5.4%).

Prevalence is lower among respondents with a high education level than with a medium or low one. Population prevalence of depressive symptoms thereby increases by the factor 2.5 (high education 5.9%; low education 14.8%, data not shown). Differences in prevalence with regard to levels of education are recorded across all age groups. Analyses that take into account age, gender and education indicate a prevalence of depressive symptoms of over one fifth (22.4%) of women with low education aged 18 to 29. Analogous analyses for men show the highest prevalence in those with low education aged 30 to 44 (17.8%). Prevalence is lowest in both genders for those aged over 65 with high education (women 3.6%; men 4.2%).

**Figure 1:**
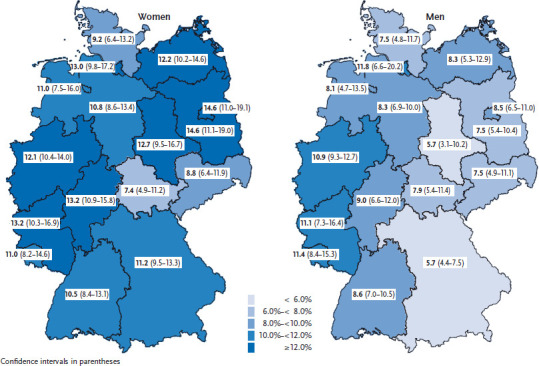
Prevalence of depressive symptoms during the past 2 weeks (PHQ-8 score ≥10) according to gender and federal state (n=12,900 women; n=10,702 men) Source: GEDA 2014/2015-EHIS

The prevalence of depressive symptoms varies between federal states (Figure 1). Women from Berlin and Brandenburg (14.6%) are nearly twice as likely to present depressive symptoms compared to women from Thuringia (7.4%). Significant differences in prevalence for men are found between Bavaria (5.7%) and Saarland (11.4%), as well as North-Rhine Westphalia (10.9%). Differences in prevalence between women and men are particularly high in Bavaria (11.2% vs. 5.7%) and Brandenburg (14.6% vs. 7.5%) and low in Saarland (11.0% vs. 11.4%) and Thuringia (7.4% vs. 7.9%).

Total prevalence (10.1%) is comparable to current findings from the US-American National Health and Nutrition Examination Survey (NHANES) and the Behavioral Risk Factor Surveillance System (BRFSS) [18, 19]. However, the new data is not consistent with data from previous surveys that were conducted in Germany. According to DEGS1 (2008-2011), the prevalence of depressive symptoms (8.1% overall, 6.1% for men) was significantly lower than the prevalence reported by the current survey [14]. An older population survey indicated an overall prevalence of 7.2%, which is even lower than the prevalence recorded by DEGS1 [20]. Whether these differences indicate a trend over time in the prevalence of depressive symptoms among adults and/or men in Germany is unclear and to clarify this question will require further comparative data, also because the PHQ version applied differed as did the surveyed age groups. In the US, however, an increase in depressive symptoms in the population has been documented since 2005 [18].

Previous surveys have already highlighted the importance of differences in prevalence between women and men [8, 14, 19]. The results highlight the importance of the age factor and are in line with the data available, according to which depressive symptoms among adults are clearly and particularly linked to younger age [12, 14, 19]. Equally, the links between depressive symptoms and education [8, 18] as well as socioeconomic status [12, 14, 21] are well known. No comparative data on the differences in prevalence between the German federal states is available. Regional differences could be related to a region’s age and social structure, but could also be related to differences in the spatial distribution of risk and protective factors. A comparison between cities and rural areas reveals different levels of prevalence, whereby the frequencies of depressive symptoms are higher in medium-sized and large cities than in small rural towns [12, 14].

The reported results can be classified in the context of available epidemiological findings on the prevalence of diagnoses of depression. GEDA 2014/2015-EHIS reports a 12-month prevalence of self-reported medical diagnoses of depression of 8.1% which is lower than the overall prevalence of depression symptoms. DEGS1 data (additional mental health module) too reveals a lower prevalence of diagnoses of depression that are recorded through standardised clinical interviews [12, 22, 23]; whereby methodological differences in the surveys need to be considered. When the definition of depressive symptoms according to PHQ is adjusted to include defining criteria for a diagnosis of depression, frequencies also drop [24]. A medical diagnosis moreover depends on patients turning to health services and is influenced by further factors such as healthcare services coverage [25].

To record depressive symptoms, the survey applied the internationally established PHQ instrument. Based on the results from GEDA 2014/2015-EHIS, we can make statements on the adult population in Germany. Identifying the particularly burdened segments of the population provides a basis to develop target groups to which to direct prevention and care. Moreover, the survey reveals relevant questions for future surveys on the prevalence of depressive symptoms, for example concerning trends over time and regional differences. The reported prevalence should be considered as conservative estimates because persons with acute or severe depression are less likely to participate in the survey [26].

## Key statements

- One in ten adults in Germany experiences current depressive symptoms.
- Women aged 18 to 29 present a particularly high prevalence of depressive symptoms (16.4%).
- A higher education translates into a lower prevalence of depressive symptoms.
- The prevalence of depressive symptoms varies between federal states.

## Figures and Tables

**Table 1:** Prevalence of depressive symptoms in the past 2 weeks (PHQ-8 score ≥10) according to gender, age and educational level (n=12,900 women; n=10,702 men) Source: GEDA 2014/2015-EHIS

Women	%	(95% CI)	Men	%	(95% CI)
**Women total**	**11.6**	**(10.8-12.4)**	**Men total**	**8.6**	**(7.9-9.4)**
**18-29 Years**	16.4	(14.5-18.6)	**18-29 Years**	9.5	(7.7-11.7)
Low education	22.4	(17.4-28.3)	Low education	13.9	(9.8-19.3)
Medium education	15.7	(13.4-18.4)	Medium education	8.3	(6.4-10.6)
High education	9.9	(7.5-13.0)	High education	7.0	(4.1-11.9)
**30-44 Years**	10.9	(9.5-12.4)	**30-44 Years**	9.4	(7.9-11.2)
Low education	16.1	(11.5-22.1)	Low education	17.8	(12.2-25.2)
Medium education	11.4	(9.6-13.4)	Medium education	10.1	(8.0-12.7)
High education	6.4	(4.8-8.6)	High education	4.9	(3.5-6.8)
**45-64 Years**	11.9	(10.8-13.1)	**45-64 Years**	9.6	(8.5-10.7)
Low education	17.5	(14.1-21.4)	Low education	15.3	(11.7-19.7)
Medium education	11.8	(10.5-13.3)	Medium education	10.5	(9.0-12.3)
High education	7.3	(6.0-8.9)	High education	5.9	(4.7-7.4)
**≥ 65 Years**	8.7	(7.4-10.1)	**≥ 65 Years**	5.4	(4.5-6.5)
Low education	11.7	(9.4-14.4)	Low education	7.1	(4.8-10.5)
Medium education	7.1	(5.4-9.3)	Medium education	5.7	(4.4-7.5)
High education	3.6	(2.2-5.7)	High education	4.2	(3.0-5.9)
**Total (women and men)**	**10.1**	**(9.6-10.7)**	**Total (women and men)**	**10.1**	**(9.6-10.7)**

CI=Confidence interval
